# [Retracted] Inhibiting proliferation and metastasis of osteosarcoma cells by downregulation of long non‑coding RNA colon cancer‑associated transcript 2 targeting microRNA‑143

**DOI:** 10.3892/ol.2023.13665

**Published:** 2023-01-09

**Authors:** Fengjiang Bi, Can Chen, Jing Fu, Lei Yu, Jia Geng

Oncol Lett 21: 265, 2021; DOI: 10.3892/ol.2021.12526

Following the publication of this paper, it was drawn to the Editor's attention by a concerned reader that the data panels showing cell invasion assay data in Fig. 4F contained a number of internal duplications, such that the same data appeared to have been selected from the same original source(s) to represent the results from differently performed experiments; furthermore, certain of these data were also strikingly similar to data appearing in different form in other articles by different authors at different research institutes. Owing to the unacceptable number of instances of data duplication and the fact that contentious data in the above article had already been published, or were already under consideration for publication, elsewhere prior to its submission to *Oncology Letters*, the Editor has decided that this paper should be retracted from the Journal. he authors were asked for an explanation to account for these concerns, but the Editorial Office did not receive a reply. The Editor apologizes to the readership for any inconvenience caused.

